# Marine subsidies change short‐term foraging activity and habitat utilization of terrestrial lizards

**DOI:** 10.1002/ece3.3560

**Published:** 2017-11-07

**Authors:** Heather V. Kenny, Amber N. Wright, Jonah Piovia‐Scott, Louie H. Yang, David A. Spiller, Thomas W. Schoener

**Affiliations:** ^1^ Department of Wildlife, Fish, and Conservation Biology University of California Davis CA USA; ^2^ Department of Biology University of Hawai'i at Mānoa Honolulu HI USA; ^3^ School of Biological Sciences Washington State University Vancouver WA USA; ^4^ Department of Entomology and Nematology University of California Davis CA USA; ^5^ Department of Evolution and Ecology University of California Davis CA USA

**Keywords:** diet shift, habitat shift, resource pulses, temporal‐response scale

## Abstract

Resource pulses are brief periods of unusually high resource abundance. While population and community responses to resource pulses have been relatively well studied, how individual consumers respond to resource pulses has received less attention. Local consumers are often the first to respond to a resource pulse, and the form and timing of individual responses may influence how the effects of the pulse are transmitted throughout the community. Previous studies in Bahamian food webs have shown that detritivores associated with pulses of seaweed wrack provide an alternative prey source for lizards. When seaweed is abundant, lizards (*Anolis sagrei*) shift to consuming more marine‐derived prey and increase in density, which has important consequences for other components of the food web. We hypothesized that the diet shift requires individuals to alter their habitat use and foraging activity and that such responses may happen very rapidly. In this study, we used recorded video observations to investigate the immediate responses of lizards to an experimental seaweed pulse. We added seaweed to five treatment plots for comparison with five control plots. Immediately after seaweed addition, lizards decreased average perch height and increased movement rate, but these effects persisted for only 2 days. To explore the short‐term nature of the response, we used our field data to parametrize heuristic Markov chain models of perch height as a function of foraging state. These models suggest a “Synchronized‐satiation Hypothesis,” whereby lizards respond synchronously and feed quickly to satiation in the presence of a subsidy (causing an initial decrease in average perch height) and then return to the relative safety of higher perches. We suggest that the immediate responses of individual consumers to resource pulse events can provide insight into the mechanisms by which these consumers ultimately influence community‐level processes.

## 1 INTRODUCTION

**Figure 1:**
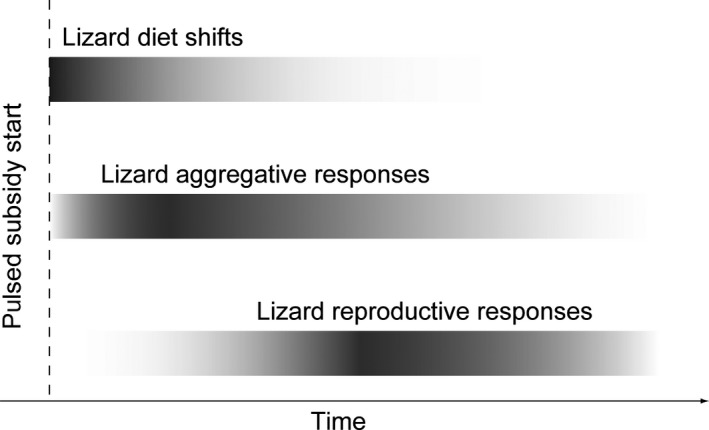
Conceptual representation of the relative importance of different lizard responses to pulsed seaweed subsidy over time. Darker regions on the gradient indicate greater relative importance. The time axis is heuristic and is not presented to scale; behavioral shifts are expected to occur within minutes, aggregation responses are expected to occur within days to weeks, and reproductive responses are typically expected to occur after several months

The ways in which consumers cope with spatial and temporal variation in resource availability have implications for ecological dynamics at the population, community, and ecosystem levels. Resource pulses—infrequent, high magnitude, ephemeral increases in resource availability—provide a natural framework for exploring these dynamics (Yang, Bastow, Spence, & Wright, 2008). Consumer responses to pulsed resources generally fall into three categories: (1) altered behavior of local consumers, (2) spatial aggregation to resources by nonlocal consumers, and (3) increased reproduction (Bergeron, Réale, Humphries, & Garant, 2011; Curran & Leighton, 2000; Epanchin, Knapp, & Lawler, 2010; Norris & Martin, 2014; Ostfeld & Keesing, 2000; Yang et al., 2008). The relative importance of these different responses is expected to change with elapsed time since initiation of the resource pulse. The behavioral responses of local consumers can be almost immediate, whereas there is generally a time lag before numerical responses due to aggregation or reproduction can occur (Yang et al., 2010). The speed of a reproductive response in particular is limited by the generation time of the consumer. Figure 1 shows how the different timescales on which these three responses occur alter their relative importance to indirect consumer effects on other ecosystem components over time. Under this conceptual model, the timing and duration of the pulse determine which responses are most likely. For example, it could be that behavioral shifts are particularly important when resource pulses are small but frequent because there is not sufficient time for aggregation or reproduction before the resource dissipates. Conversely, we would expect the individual behavioral response to be relatively less important than aggregation or reproduction when resource pulses are larger in magnitude and longer in duration. There is theoretical support for the idea that the relative timescales of aggregation and reproduction influence consumer‐mediated effects of allochthonous resource inputs on in situ resources (Takimoto, Iwata, & Murakami, 2009). However, our understanding of how consumers respond across multiple timescales remains incomplete without integrating the short‐term behavior of individuals.

Two key ways that resident consumers can rapidly respond to resource pulses are to change what they eat and where they eat, and these individual responses can translate into effects at the community level. Opportunistic generalist consumers often alter their diets to capitalize on ephemeral resources (Schmidt & Ostfeld, 2008; Yang et al., 2008), which can result in a range of community‐level effects that can persist over longer time periods (Abrams & Matsuda, 1996; Polis, Anderson, & Holt, 1997). For example, pulses of rodents are associated with short‐term positive effects on birds due to diet switching by generalist predators, followed by high predation rates on birds in years when rodents crash (Schmidt & Ostfeld, 2008). Changing diet to take advantage of pulsed resources may also require changing foraging behavior and habitat use (McLoughlin, Lysak, Debeffe, & Perry, 2016). Shifting habitat use by consumers could contribute to a behavior‐mediated cascade where herbivory levels change because prey are released from predation in certain parts of the habitat (Beckerman, Uriarte, & Schmitz, 1997; Schmitz, 2005; Schmitz, Beckerman, & O'Brien, 1997; Werner & Peacor, 2003). At the same time, habitat shifts by consumers may be constrained by trade‐offs with their own predation risk (Schmitz, Krivan, & Ovadia, 2004; Werner & Peacor, 2003). Here, we investigated shifts in habitat use and foraging behavior in lizards known to undergo a diet shift in response to resource subsidies.

The effects of pulsed resources have been studied extensively in shoreline ecosystems in the Bahamas. These ecosystems have relatively simple food webs consisting of arthropod herbivores, arthropod predators (such as spiders), and vertebrate predators. The most common vertebrate predator is the lizard *Anolis sagrei*. Pulses of allochthonous marine resources enter these ecosystems in the form of seaweed deposits, which frequently occur in association with storms. These pulsed seaweed subsidies alter the structure and dynamics of recipient food webs. When seaweed is abundant, lizards undergo a diet shift from eating primarily terrestrial prey to eating more marine‐derived prey (i.e., seaweed detritivores), as shown by stable isotope analysis (Spiller et al., 2010). In addition to the functional response, lizards also respond numerically by aggregating into seaweed‐subsidized areas and growing faster, the latter of which likely increases lifetime reproductive success (Wright et al., 2013). When lizards shift to alternative prey, terrestrial arthropod abundance and herbivory levels increase, followed by decreases in terrestrial arthropod abundance when the pulse recedes and lizards switch back to eating terrestrial prey (Piovia‐Scott, Spiller, & Schoener, 2011; Piovia‐Scott et al., 2013; Spiller et al., 2010). Here, we complement previous work in this system by more closely investigating the short‐term response of lizards to seaweed pulses.

We manipulated seaweed abundance in shoreline plots and compared lizard perch height, rate of attacks on prey when foraging, and movement activity between seaweed addition plots versus control plots. *Anolis* lizards provide a good model for exploring behavioral responses to resource variation as they have been extensively studied and many aspects of their biology and ecology are well understood (see Losos, 2009). For example, perch height varies depending on sex (Lister & Garcia Aguayo, 1992; Schoener, 1967, 1968), predation risk (Scott, Wilson, Jones, & Andrews, 1976), abiotic conditions (Lopez‐Darias, Schoener, Spiller, & Losos, 2012), and hunger level (Paterson, 1999; Stamps, 1977; Stamps & Tanaka, 1981) and is therefore a good metric to evaluate anole foraging behavior and habitat use. Like many other anoles (Stamps, 1977), *A. sagrei* is typically regarded as a sit‐and‐wait forager that scans the ground for invertebrate prey from arboreal perches (Schoener, 1968, 1979). Sit‐and‐wait foraging in particular requires a balance between using high perches which provide a better vantage point and more safety from predators such as curly tail lizards (*Leiocephalus carinatus,* which occur in our study plots), and using low perches which allow for greater capture success rate on ground‐inhabiting prey (Scott et al., 1976). Therefore, we predicted that lizards would decrease their perch height and increase their attack rate as they shifted to more actively foraging for abundant prey in seaweed deposits on the ground. Also, we expected increased movement if lizards are shifting from a sit‐and‐wait strategy to more active foraging in the presence of abundant food resources. Finally, we used field data from this study to parameterize a heuristic model linking lizard activity and perch height in order to examine how pulsed resources could influence the habitat use of lizards by synchronizing foraging behavior and satiation responses.

## 2 MATERIALS AND METHODS

### 2.1 Study system

This study was conducted on five islands near Great Abaco, Bahamas, in September 2013. On each island, two 5 × 10 m plots were established and randomly assigned as control (no seaweed added) or treatment (seaweed added). Each pair of plots was considered to be a block in statistical analyses because they occurred on the same island and were within 100 m of each other. Thus, our design included *n* = 5 control and *n* = 5 treatment plots, and plots were paired on five islands that served as blocks.

### 2.2 Lizard observations

Lizard observations were conducted by one individual (HVK) 12–19 September 2013 and occurred between the hours of 0845 and 1745 each day. For each observation, the first undisturbed lizard (i.e., the lizard did not move as it was approached) that was spotted in a plot was followed and filmed continuously from no closer than 1 m using a Canon VIXIA HF R400 camcorder for up to 20 min or until the lizard moved out of sight. The lizard's movements and location were visually estimated in centimeters and narrated as they occurred. Each time the lizard moved, the total distance traveled (horizontal plus vertical) was estimated to capture the full extent of the movement, as well as the new perch height. Each bite observed was recorded and assumed to be a foraging attempt (hereafter attack). In the treatment plots after the seaweed was added, the distance to the nearest edge of the seaweed pile was recorded for each perch. Distance to seaweed was measured as horizontal distance; if a lizard was perched on a branch directly above the seaweed, it was scored as zero. After returning from the field, videos were scored and the narrated values were recorded in a spreadsheet for data analysis. Any behaviors that were overlooked during the initial video recording (such as additional attacks) were scored along with the narrated observations.

In order to determine the degree to which individuals were repeatedly measured over time, we attempted to capture and mark lizards immediately following an observation period or during nonobservation visits. The sex and snout‐vent length (SVL) of captured lizards were recorded, and they were uniquely marked with dots of nontoxic paint applied to the dorsum. If the lizard was not captured, it was marked with diluted nontoxic paint using a small spray bottle. As not all observed individuals could be caught and measured, their size was estimated and split into two size classes: large (SVL ≥ 30 mm) and small (SVL < 30 mm).

### 2.3 Experimental seaweed subsidies

Seaweed was collected from local beaches and deposited in a berm along the length of each treatment plot above the high tide line. Subsidized plots received 2.5 kg of seaweed per square meter of vegetated area (between 72.5 and 122.5 kg per plot), which is consistent with the amount deposited during natural seaweed deposition events (Spiller et al., 2010). Seaweed was added to three of the seaweed addition plots on 16 September 2013 and to the remaining two plots the following day. A buffer time of at least 20 min was allowed between the time the seaweed subsidy was finished being deployed and the start of an observation in that plot. Observations occurred from 122.8 to 0.27 hrs before subsidies were applied and from 0.35 to 72.9 hrs after subsidy application.

### 2.4 Data analysis

To test whether lizard behavior changed following seaweed addition, we used generalized linear mixed models to compare behaviors (mean perch height, number of attacks, and number of moves) between control and treatment plots as a function of time since subsidy. All models were fit using lme4 in R version 3.0.2 (Bates, Maechler, & Bolker, 2014; R Core Team 2013), and hypothesis tests were conducted using likelihood ratio tests with an alpha of 0.05. For seaweed addition plots, time since subsidy for each observation bout was measured as minutes since seaweed was added to each plot. For control plots, the time since subsidy was measured as minutes since seaweed was added to the seaweed addition plot in the same block. Treatment, time since subsidy and a treatment by time since subsidy interaction were included as fixed effects, and plot and block were included as random effects in all models. We evaluated changes in response over time by comparing confidence intervals on model fits. To account for pre‐existing differences among plots, we used the mean value of the respective response variable from observations conducted before seaweed addition as a plot‐level covariate. For the analysis of mean perch height, a time‐weighted mean perch height was calculated for each bout by taking the mean of perch heights used weighted by time spent at each height (including the ground, where perch height is 0). Both the number of attacks and number of moves were modeled using a Poisson generalized linear mixed model with the log of the duration of observation bout as an offset. This is a standard approach for analyzing rate data where the response is a count and the offset is the interval over which the count data were collected (Crawley, 2007).

For the response variables, mean perch height, number of attacks, and number of moves, we included the factor stage (small or large) in separate analyses. Sampling within stage categories after subsidy application was uneven (control: large 3, small 12; treatment: large 13, small 11), and we did not have enough data to fit models that also included time since subsidy as a predictor. Instead, we included a treatment by stage interaction to test whether large versus small individuals responded differently to subsidy.

In seaweed addition plots, we evaluated the relationship between lizard perch height and distance to seaweed. We used a linear mixed model with mean perch height in each observation as the response variable, distance from seaweed as a continuous predictor, and plot as a random factor.

### 2.5 Modeling synchronized satiation

We modeled lizard perch height as a function of activity state using Markov chain models parameterized from field data. In these heuristic models, lizards exist in one of two states: actively foraging (i.e., moving) or not actively foraging (i.e., sit and wait). Transitions between these two states define a 2 × 2 Markov matrix *P*, where each entry *p*_*ij*_ is the probability that a lizard is in state *i* after it was in state *j* (Fig. S1). In the baseline model, actively foraging lizards have a 55% probability of remaining active and a 45% probability of becoming inactive at each time step. Inactive lizards have a 95% probability of remaining inactive and a 5% probability of becoming active at each time step (Appendix S1). This transition matrix was designed to result in an expected steady state where lizards spend 10% of their time foraging, qualitatively consistent with field observations in this and other studies (reviewed in Losos, 2009). The perch height for each lizard in the model is randomly drawn from state‐dependent gamma distributions. These distributions were parameterized from the observed perch heights of control lizards separately for active and inactive states, with active lizards perching lower than inactive lizards (Table S1, Appendix S2). The gamma distribution was chosen to flexibly represent the observed (and non‐negative) distribution of perch heights. We defined the active state as greater than 0.8 moves per minute based on a break in the observed distribution of moves per minute (Fig. S2). This threshold is supported by Cooper (2005), and other thresholds showed qualitatively similar patterns.

We used this model structure to test the effect of synchronized responses of multiple individual lizards to the subsidy, the effect of increased satiation in response to subsidy, and the combined effects of these factors. The resource pulse was modeled as a perturbation whereby lizards undergo a behavioral shift. Four models were evaluated: (1) the “control model” using the baseline transition matrix with no perturbation, (2) the “synchrony model” using the baseline transition matrix perturbed by a single time step simulated resource pulse event in which 90% of lizards initiated active foraging, followed by an immediate return to the baseline transition matrix, (3) the “satiation model” using the baseline transition matrix perturbed with a simulated resource pulse event which did not synchronize the activity states of the population, but instead changed the transition matrix to yield a steady state with reduced foraging activity (5% actively foraging, 95% not actively foraging, Appendix S1) for the remainder of the simulation, and (4) a “synchrony and satiation model” using the baseline transition matrix perturbed by a single time step simulated resource pulse event in which 90% of lizards initiated active foraging, followed by the same “reduced foraging” transition matrix as in the satiation model.

All simulations were run for 100 time steps with a population of 500 individuals using the markovchain package in R version 3.1.1 (R Core Team, 2013; Spedicato, 2015). The perturbation was imposed at time step 50 in order to generate both pre‐ and postperturbation perch heights. Initial activity states for the population were drawn randomly from the steady‐state expectation for the baseline model. At each time step, the mean perch height across all individuals was calculated and saved for visualization. In addition to the numerical simulations, the expected mean perch height was calculated analytically for each set of model conditions using the Chapman–Kolmogorov equation.

## 3 RESULTS

We recorded a total of 87 video observations with mean observation length of 15.4 ± 5.6 min (mean ± *SD*). Data are available from the Dryad Digital Repository (doi:10.5061/dryad.bc0qk). Data taken before subsidy (*n* = 48 observations) were averaged at the plot level over the entire presubsidy period to provide baseline measures of behavior (4.8 ± 1.9, mean ± *SD* observations per plot).

Some lizards were observed more than once. Based on our marking efforts, we were able to determine that postsubsidy, 23 of the 39 observations were of marked individuals that were only observed once, three marked individuals were observed twice (six observations total), and 10 observations were of unmarked animals. Observations of unmarked animals occurred in five plots (two control, three treatment), with each plot having two observations of unmarked juveniles. For the plots with unmarked juveniles, it is possible that we observed two different unmarked juveniles or the same juvenile twice. However, the unit of replication in this experiment is the plots because treatments were applied at the plot level; lizards within plots are subsamples. In our statistical models, we included a random effect for plot, which accounts for the fact that observations within a plot may be similar to each other, either because multiple lizards are affected by the same unmodeled conditions, or because an individual lizard was observed more than once.

**Figure 2:**
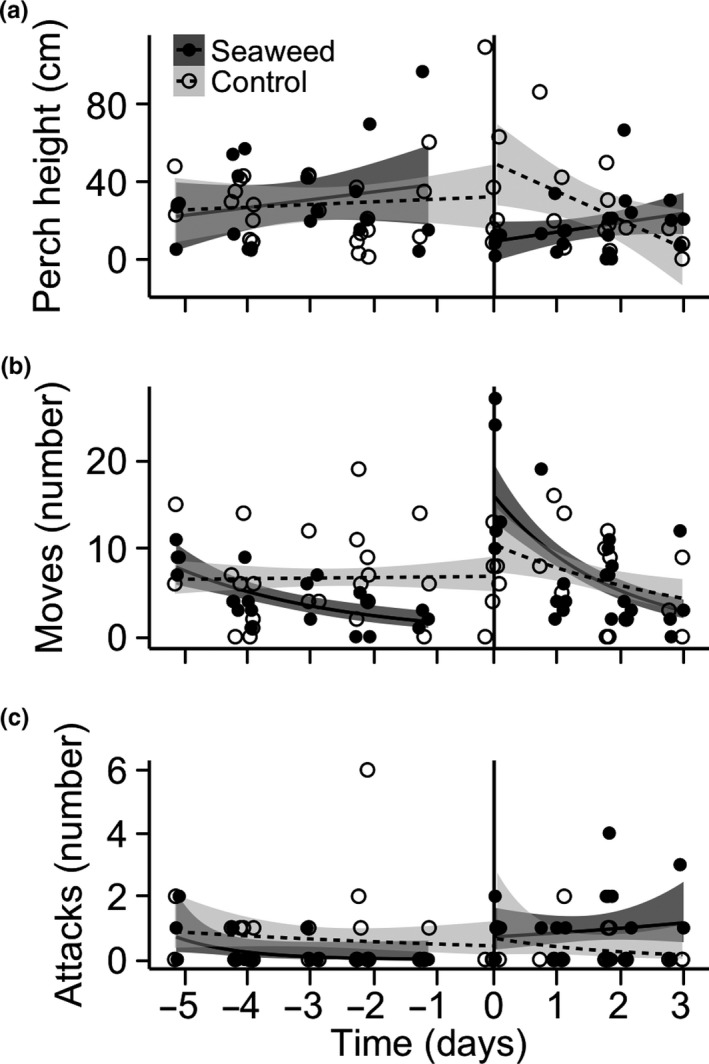
Lizard behavioral responses to seaweed addition: (a) perch height, (b) moves, and (c) feeding attacks. Filled symbols and dark shading are seaweed addition plots, and open symbols and light shading are control plots. Each point represents a lizard observation, smooth curves and standard errors are from generalized additive models fit to the data, and the vertical line represents the time when seaweed was added to treatment plots. For (b) moves and (c) feeding attacks, generalized additive models featured Poisson errors and a log link function

**Figure 3:**
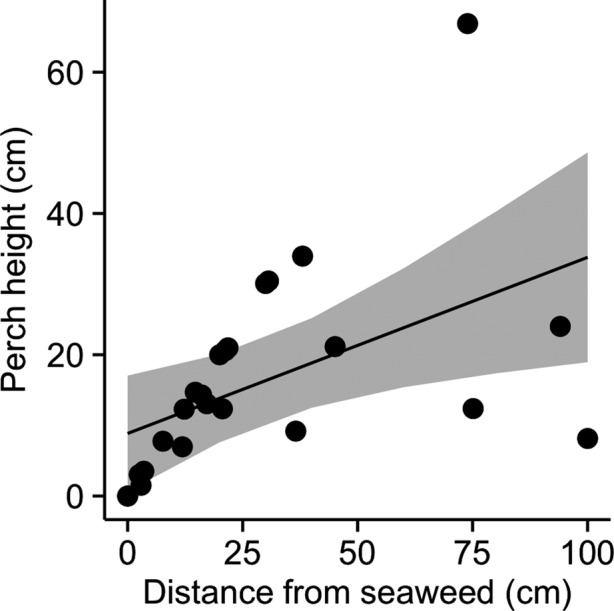
Lizard perch height and distance from seaweed in plots to which seaweed was added. Line and shaded area represent best fit and standard errors from the model described in the text

**Figure 4:**
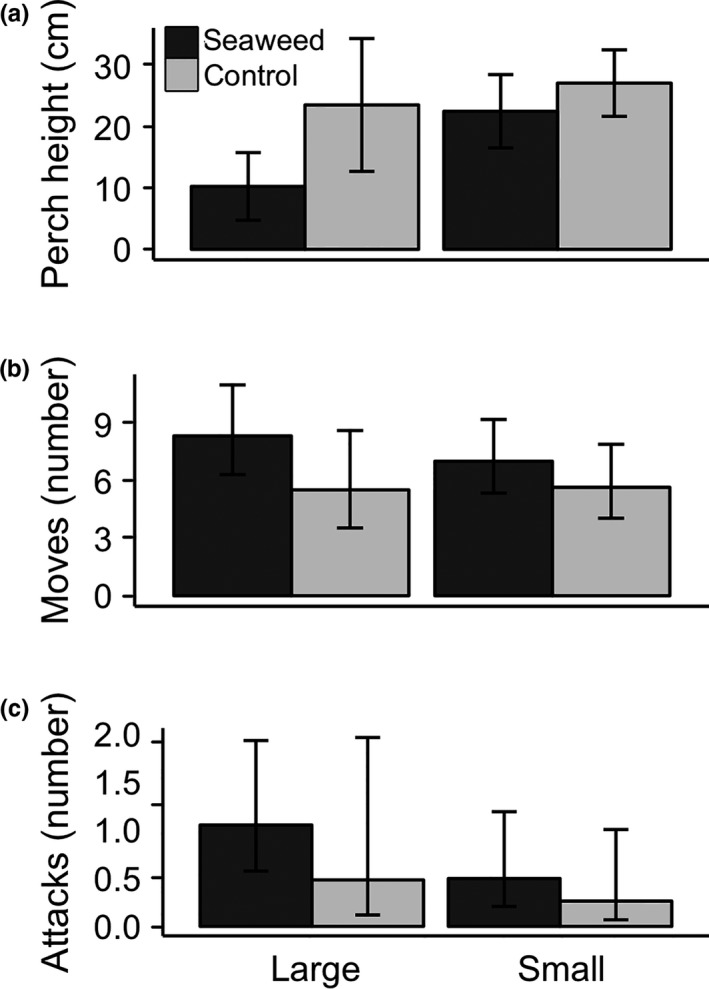
Lizard behavioral responses following seaweed addition for small (<30 mm) versus large (≥30 mm) lizards: (a) perch height, (b) moves, and (c) feeding attacks. Data represent least‐square means and standard errors for observations conducted after seaweed deposition from the model described in the text

Lizards perched lower in treatment plots immediately following seaweed addition (treatment × time since subsidy χ^2^ = 11.33, *p* = .0008; Figure 2a, Fig. S3a), but based on confidence intervals around the model fit, mean perch height was similar to control values 2 days after seaweed addition. In addition, lizards perched lower to the ground when they were closer to seaweed in subsidized plots (χ^2^ = 6.4, *p* = .01; Figure 3). Animals in treatment plots moved more than control animals immediately following seaweed addition, and this difference decreased over time (treatment × time since subsidy χ^2^ = 6.95, *p* = .008; Figure 2b, Fig. S3b). There was no difference in the number of attacks between treatment and control plots (treatment χ^2^ = 0.27, *p* = .60; Figure 2c, Fig. S3c). No differences were detected between small and large individuals in response to subsidy (treatment × stage: perch height χ^2^ = 0.39, *p* = .53; number of attacks χ^2^ = 0.02, *p* = .90; number of moves χ^2^ = 0.40, *p* = .53; Figure 4), although uneven replication across life stages limited our power to detect these differences.

**Figure 5:**
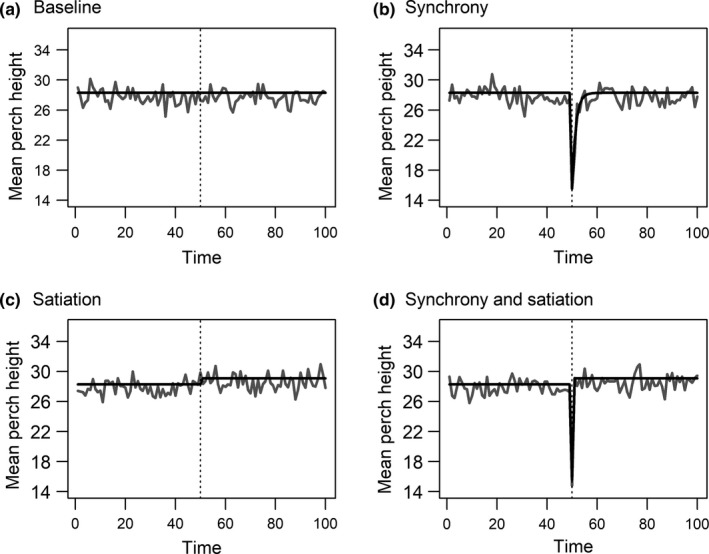
Mean perch height over time from heuristic Markov chain models of lizard foraging activity parameterized from field data (*n* = 500 simulated lizards). The dashed line represents the subsidy perturbation at time 50. Gray lines are numerically generated model predicted mean perch heights. Black lines are analytically generated model predicted mean perch heights. a) Baseline model with no perturbation, b) Synchrony model: 90% of lizards switch to active foraging for 1 time‐step following perturbation, c) Satiation model: lizards forage less after perturbation, d) Synchrony and Satiation: combined synchrony and satiation conditions. See main text for additional model details

The model simulations showed different patterns of perch height depending on the inclusion of synchrony and/or satiation. Relative to the baseline simulation (Figure 5a), a synchronized consumer response to the subsidy caused a transient reduction in the population mean perch height lasting for approximately five time steps (Figure 5b). This transient reduction occurred even though there was no change in the underlying transition matrix (i.e., no reduced foraging due to satiation after subsidy; Figure 5b). Without a synchronized consumer response, a reduction in foraging activity (i.e., satiation) leads to a slight increase in the population mean perch height (Figure 5c). Combining a synchronized response with increased satiation following the prey subsidy yields a pattern of transiently decreased population mean perch height of approximately one time step, followed by a slight increase relative to baseline conditions (Figure 5d).

## 4 DISCUSSION

We observed rapid responses by lizards to experimental resource pulses. Lizards in treatment plots used lower average perch heights immediately after the initiation of the subsidy, a change in habitat‐use behavior that may allow these consumers to better capitalize on the pulsed food resource. Lizards also showed increased movement rates immediately after the subsidy, which suggests that a change in time allocation to different behaviors accompanied the change in perch use. Both of these responses were rather short term, lasting for approximately 2 days postsubsidy.

Terrestrial vertebrate predators foraging in seaweed wrack is a common feature of shoreline ecosystems (Colombini, Chelazzi, Gibson, & Atkinson, 2003; Dugan et al. 2003; Kirkman & Kendrick, 1997; Rose & Polis, 1998; Stewart, Herman, & Teferi, 1989). Several of the observed responses in the current study were consistent with lizards foraging for marine‐derived prey in seaweed wrack, as documented previously in this system by Spiller et al. (2010) based on stable isotope analysis. A decrease in the average perch height of the lizards indicates that there was a rapid shift in habitat use consistent with foraging in seaweed on the ground in subsidized plots. This result is similar to the habitat shift recorded by Lister and Garcia Aguayo (1992), where *Anolis nebulosus* responded to seasonal variation in arthropod density by increasing average perch height when prey availability was higher in the canopy. In our study, the shift toward the ground was accompanied by an increased movement rate—instead of spending most of their time at a single perch, subsidized lizards were more frequently moving along the ground or between the ground and the vegetation. Previous research on anoles and other lizard species has also demonstrated similar changes in foraging strategy from sit‐and‐wait to more active search (e.g., Greeff & Whiting, 2000; Lister & Garcia Aguayo, 1992) which together allows individuals to exploit a wider range of food resources. We interpreted these two rapid responses—a decrease in average perch height and an increase in movement rate—as evidence for a substantial shift in foraging and habitat use by lizards in response to the prey subsidy.

However, we did not see an increase in attack rate, which we defined as the number of observed bites (assumed to be foraging events) over time, in response to subsidy. We expected attack rate to increase because previous studies have shown that prey was more abundant postsubsidy, subsidized lizards showed an increased marine signature in their diet, and subsidized lizards grew faster (Spiller et al., 2010; Wright et al., 2013). It is possible that we were unable to detect a change in attack rate with our methods because prey captures are rarer and more stochastic events than perch use or movements. With our approach, every lizard seen could be scored for perch use and movement, but most (64%) focal observation bouts did not capture any foraging events. This may be addressed in future studies using longer observation bouts of more individuals. Alternatively, if the percentage of attacks that result in successful prey capture is higher in subsidized plots (e.g., because marine‐derived prey are easier to capture than terrestrial prey on average), then foraging in seaweed would not necessarily be associated with increased attack rate. Finally, it is possible that the observed shift in habitat use and activity was due to exploratory behavior by lizards in response to a novel stimulus (i.e., the addition of seaweed) (Lapiedra, Chejanovski, & Kolbe, 2017). Given that we have previously documented diet shifts in response to subsidy, we suspect that both exploratory and foraging responses are likely involved in the habitat and activity shift.

While we predicted that lizards would reduce their perch height to take advantage of added seaweed resources on the ground, we did not expect the response to be so short‐lived. We used a model of perch height as a function of foraging state to explore two plausible mechanisms that could explain the short duration of changes in lizard perch height: (1) a rapid, synchronized foraging response (i.e., “synchrony”) immediately following the pulsed subsidy event and (2) more persistent increases in the availability of prey which reduced the steady‐state time spent foraging postsubsidy (i.e., “satiation”). Our analysis of these models suggested that the observed transient reduction in population mean perch height is consistent with a synchronized consumer response in which a larger than usual fraction of the population shifts to active foraging immediately after subsidy. This could be because multiple individuals are responding simultaneously to the resource pulse when it first appears. In contrast, our simulations found that satiation, or reduced foraging in the presence of subsidy, led to a slight increase in perch heights. Combined synchrony and satiation results in both a transient reduction and slight long‐term increase in perch height. Longer‐term observations would be necessary to determine whether lizards show increases in perch height above baseline in our system, but the pattern of initial perch height reduction followed by perch height increase has been documented in *Anolis* lizards before. Stamps (1977) placed ad libitum prey on the ground and found that *Anolis aeneus* came down from the vegetation, fed to satiation, and then climbed back up to greater perch heights than used immediately before feeding. Paterson (1999) observed that female *Anolis distichus* initially perched lower on average in response to experimental food subsidies in their home range but then increased their average perch height above the presubsidy average 24 hrs postsubsidy.

Two alternative explanations for the transient nature of the behavioral response are that lizards returned to higher perches because the availability of seaweed detritivores (mainly amphipods) declined, and/or the lizards have a lagged response to a major terrestrial predator (curly tail lizards, *L. carinatus*) of our focal consumer (*A. sagrei*). Amphipod availability could decline because they are locally depleted by predators or because they move from the surface of the seaweed pile to the interior to avoid desiccation. In a previous experiment (Spiller et al., 2010), amphipod biomass was much higher in subsidized plots several weeks after the initial subsidy and peaked several months postsubsidy, although seaweed was added repeatedly over time compared to a single addition in this study. This relatively long interval of elevated amphipod availability suggests that local depletion is not occurring over a few days, but daily measurements of amphipod abundance would be necessary to rule it out. We do not think that the desiccation explanation is likely because it rained repeatedly during the days that postsubsidy behavioral observations were conducted. In terms of a potential lagged effect of curly tail lizards on *A. sagrei*, it is possible that the latter are able to rapidly take advantage of pulsed resources, but that this behavior becomes riskier over time if larger predators respond more slowly to the subsidy.

The rapid shifts in foraging activity and habitat use observed in this study are consistent with the population‐ and community‐level effects of pulsed subsidies observed in previous studies. While this short‐term study captured the initial potentially synchronized response of lizards to exploit seaweed‐derived resources, this short‐lived response alone likely would not cause the previously observed (Spiller et al., 2010) change in isotope signature. It seems likely that lizards also undergo a broader shift in their foraging strategy. For example, if lizards continually make short forays to feed on the ground (as opposed to a sustained shift in habitat use), this could result in an overall major diet shift toward consuming more marine prey. Under the synchronized‐satiation hypothesis, lizards would spend more time higher in the vegetation and less time on the ground, which could help confer protection from predators. *Anolis sagrei* has been shown to move up into the vegetation when larger, predatory lizards are present on the ground (Lopez‐Darias et al., 2012; Losos et al., 2006; Schoener, Spiller, & Losos, 2002). While reproductive and aggregative responses to subsidy have been shown to increase lizard density (Spiller et al., 2010; Wright et al., 2013), reduced risk of predation may also contribute directly to *A. sagrei* population growth through increased survival or indirectly through reduced stress (e.g., Schoener & Spiller, 2012; Werner & Peacor, 2003).

The addition of pulsed resources to an ecosystem can have numerous effects, from very fine scale individual behavior to changes in population and food web dynamics. In this study, we show that lizards changed their habitat use and movement patterns in response to pulsed seaweed subsidies. The hypothesized mechanism of repeated short feeding bouts (which are synchronized when the resource pulse first appears) as opposed to a sustained habitat shift provides a new perspective for examining consumer responses to resource variability. Further research on behavioral responses to resource pulses has the potential to provide additional insights into the linkages between individual‐, population‐, and community‐level responses to perturbation.

## CONFLICT OF INTEREST

None declared.

## AUTHOR CONTRIBUTIONS

HVK, ANW, JPS, LHY, DAS, and TWS conceived and designed the study. HVK conducted the fieldwork and collected the data. HVK, ANW, JPS, and LHY analyzed the data. LHY developed the mathematical models. HVK and ANW wrote the manuscript; all authors edited the manuscript.
