# Supplementary material for: Marine subsidies change short‐term foraging activity and habitat utilization of terrestrial lizards
Source: Ecol Evol. 2017 Nov 7;7(24):10701–9. doi: 10.1002/ece3.3560 (PMC5743576; doi:10.1002/ece3.3560)
Supplement: Supplementary file 1 [file ECE3-7-10701-s001.docx]

**Supporting Information**

Marine subsidies change short-term foraging activity and habitat use of lizards

Heather V. Kenny^1^, Amber N. Wright^2*^, Jonah Piovia-Scott^3^, Louie H. Yang^4^, David A. Spiller^5^, Thomas W. Schoener^5^

^1^ Department of Wildlife, Fish, and Conservation Biology, University of California, Davis

2 Department of Biology, University of Hawai'i at Mānoa

^3^ School of Biological Sciences, Washington State University, Vancouver

^4^ Department of Entomology and Nematology, University of California, Davis

^5^ Department of Evolution and Ecology, University of California, Davis

**Figure S1:** Conceptual model of lizard foraging activity with transitions between actively foraging and not actively foraging behavior states.

We investigated alternative hypotheses for the transient reduction in perch height observed in this study using Markov chain models. The models include two transition states: actively foraging and not actively foraging, as shown in the conceptual figure below.


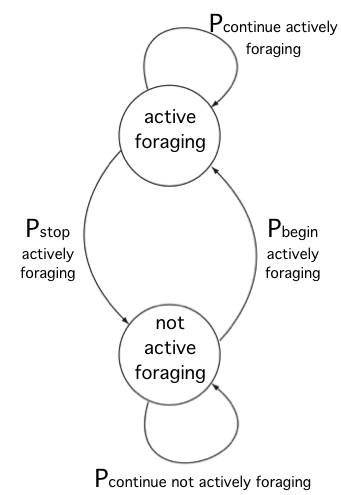


**Appendix S1:** Transition matrices for the Markov chain models

The model shown above defines a 2x2 transition matrix that gives the probability of changing foraging state at each time step.

$$P=\left[ \begin{matrix} p_{continue actively foraging} & p_{stop actively foraging} \\ p_{begin actively foraging} & p_{continue not actively foraging} \end{matrix} \right]$$

The baseline transition matrix (*P_baseline_*) results in a steady state expectation where 10% of the population is expected to be actively foraging at any given time.

$$P_{baseline}=\left[ \begin{matrix} 0.55 & 0.45 \\ 0.05 & 0.95 \end{matrix} \right]$$

A second transition matrix (*P_satiation_*) was used in some simulations in order to simulate the expectation of reduced active foraging after the introduction of the seaweed subsidy. The following matrix was used to represent the reduced foraging state.

$$P_{satiation}=\left[ \begin{matrix} 0.05 & 0.95 \\ 0.05 & 0.95 \end{matrix} \right]$$

**Table S1:** Perch height as a function of activity and presence of seaweed. Active lizards perch lower than inactive lizards whether seaweed is present or not.

**Appendix S2:** Fit of perch height data to gamma distribution

The gamma density function (Evans et al. 2000)

$$f\left( x;k,\theta\right)=\frac{x^{k-1}e^{-x/\theta}}{\theta^{k}\Gamma(k)}$$

was fit with the shape parameter *k* set equal to

$$\left( \bar{P}/s_{P} \right)^{2}$$

and with the scale parameter $\theta$ set equal to

$${s_{P}}^{2}/\bar{P}$$

where $\bar{P}$ is the mean of the mean perch heights from each observation, and *s_P_* is the standard deviation of these means. A separate gamma distribution was fit for active and inactive observations without seaweed. ECDF plots of perch height data overlain with model fits are shown below for a) inactive and b) active lizards in the absence of seaweed subsidy.

**Figure S2:** Histogram of observed moves per minute. Cut-off for active vs. inactive was 0.8 moves per minute.

**Figure S3.** Lizard behaviors in treatment and control plots after seaweed addition: A) perch height, B) moves, and C) attacks. Each point represents a lizard observation, fits and 95% confidence intervals from linear models described in the methods are shown.
